# A new route towards polarized luminescence: 0D/2D nanocomposites

**DOI:** 10.1038/s41377-023-01370-5

**Published:** 2024-01-31

**Authors:** Andries Meijerink

**Affiliations:** 1 Debye Institute for Nanomaterials Science, Department of Chemistry, Utrecht University, Princetonplein 1, 3584 CC Utrecht, The Netherlands; 2 School of Materials and Energy, Lanzhou University, Lanzhou, Gansu China

**Keywords:** Nanoparticles, Liquid crystals

## Abstract

Combining wide bandgap 2D inorganic materials and blue-light-emitting 0D carbon dots in 0D/2D heterojunction nanocomposites was shown to give rise to unique optical properties and a multifunctional prototype device was developed, capable of polarized light luminescence, modulation and detection.

The polarization of light refers to the direction of oscillation for the electric field vector of a propagating light wave. Fluorescent molecules or materials can emit light with specific polarization characteristics. The phenomenon was initially observed and documented almost a century ago by Perrin in 1926 in fluorescent molecular solutions^1^. Materials demonstrating this physical phenomenon generate diverse interests ranging from purely scientific explorations to incorporation in devices, including 2D and 3D displays, optical data storage, optical biosensors and material structure analysis^2–5^. Clearly, the advancement of polarized fluorescent materials with high efficiency and stability has great significance in both fundamental science and practical applications.

In the past decades different zero-dimensional (0D) nanosized emitters have been discovered, including quantum dots and carbon dots. Quantum dots are semiconductor nanocrystals consisting of several hundreds to thousands of atoms and have emerged as a novel class of luminescent materials which was recently recognized by the Nobel Prize of Chemistry. Owing to pronounced quantum confinement effects, quantum dots exhibit remarkably size-tunable emission colors with high fluorescence quantum efficiency and color purity^6,7^. More recently another class of nanosized 0D emitters evolved, known as carbon dots, where a conjugated π-system gives rise to efficient light emission. Conventional quantum and carbon dots are typically characterized by geometric and optical isotropy, resulting in the predominant emission of non-polarized light. To some extent, it restricts their application in the field of polarized optics. Consequently, attaining efficient and polarized luminescence based on a 0D nanosized emitter represents a pivotal frontier challenge in the advance of luminescent materials and devices.

Recently, a collaborative effort involving the teams of Baofu Ding and Hui-Ming Cheng from the Shenzhen Institute of Advanced Technology (SIAT), Chinese Academy of Sciences (CAS), Bilu Liu from the Shenzhen Research Institute of Tsinghua University, and Andre Geim from the University of Manchester has resulted in a series of groundbreaking studies on 2D wide bandgap inorganic material based liquid crystals. These studies are rooted in the intrinsic extremely large geometric anisotropy, as well as the optical and magnetic anisotropy of these 2D materials^8–11^. The collaboration has successfully achieved precise control of optical polarization states at ultra-low fields, significantly increasing the sensitivity of liquid crystal molecules in response to a magnetic stimulus by almost three orders of magnitude. They coined the term “Giant magneto-birefringence effect” and pioneered the discovery of the phenomenon of transmissive magneto-induced interference colors, and established a new research direction of highly responsive 2D wide bandgap liquid crystals that can be used to probe magnetic field strengths by monitoring color changes^8^.

Based on their previous research, Baofu Ding, Feng Wang and Hui-Ming Cheng, recently published a paper in “Light: Science and Application”^12^. They proposed a concept called “Dimensional Marriage” to graft 0D blue emitting carbon dots onto 2D cobalt-doped titaniumdioxide (Co-TiO_2_) nanosheets, creating a 0D/2D carbon dot/nanosheet heterojunction luminescent systems (Fig. 1). Aligning the highly anisotropic (nm thickness, μm lateral size) 2D sheets resulted in a nanocomposite that combines a high blue-luminescence efficiency and specific polarization, offering a novel approach for realizing a new class of polarized luminescent materials. The construction of the 0D/2D heterostructures relies on effectively anchoring 0D carbon dots by inducing the formation of Ti-O-C bonds through chemical adsorption. The combination of the high luminescence efficiency of 0D unpolarized-luminescent nanomaterials with the potent optical polarization modulation capability of 2D non-luminescent materials creates a new 0D/2D nanocomposite achieving polarized and highly efficient luminescence. Additionally, by taking advantage of the heterojunction’s wavelength dependent dichroic absorption, detection of ultraviolet light in the range of 360–385 nm was achieved. This led to the construction of a multifunctional optoelectronic device integrating polarized blue light emission, ultraviolet light detection, and visible light modulation.

**Fig. 1:**
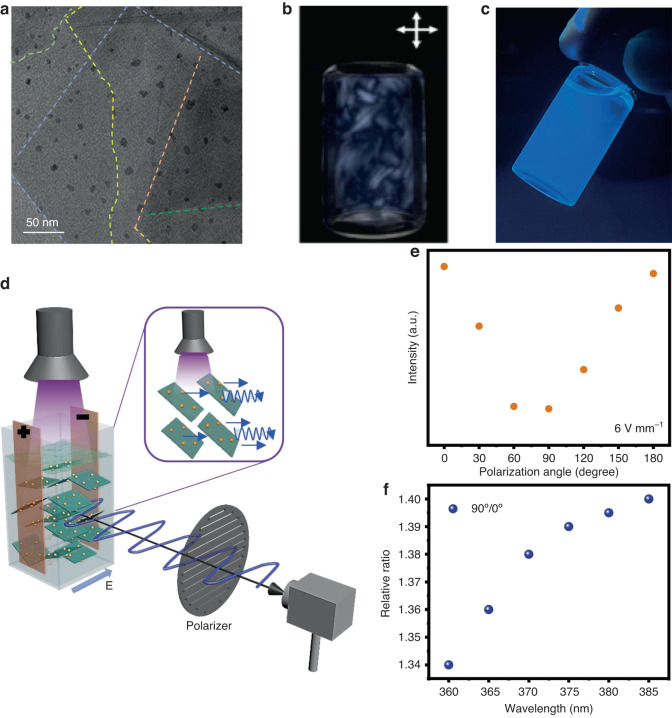
Polarized luminescence from a 0D/2D nanocomposite. **a** TEM image of inorganic 0D carbon dot/2D nanosheet heterojunction. **b** Birefringent pattern for a dispersion of 0D carbon dot/2D nanosheets in the presence of crossed polarizers. **c** Photograph under 365 nm light illumination. **d** Schematic diagram of working principle of multifunctional opto-electronic device where the 2D sheets are aligned in an external electric field. **e** Fluorescence polarization characteristics. **f** Linear dependence between the relative ratio of transmittances at polarizer angles of 90^o^ and 0^o^ with wavelength of heterojunction dispersion in an electric field of 6 V mm^−1^

The work introduces the concept of 0D/2D heterostructure polarized luminescent nanocomposite materials and marks a milestone in the field of polarized luminescence. The challenge of achieving efficient and polarized luminescence based on a 0D nanomaterial has been overcome ingeniously and expands the family of polarized luminescent materials. Furthermore, the construction of a multifunctional device that integrates light emission, modulation, and detection introduces new opportunities for the development of low-energy consumption, intelligent, or integrated optical devices. At the same time the remarkable findings stimulate further research into better understanding the interaction between the 0D emitters and the 2D nanosheets that gives rise to a high degree of polarization of the emission. The demonstration of efficient polarized light emission by the 0D/2D heterojunction is impressive but it will be crucial to investigate the role of e.g. the size/anisotropy, composition, refractive index and doping of the 2D nanosheets on the degree of polarization and to gain insight in the physics underlying the polarized emission. At the same time, it will be a challenge to graft other nanosized 0D emitters on 2D nanosheets. Especially semiconductor quantum dots with their size-tunable narrow band emission are promising candidates. Narrow band emission (narrower than for carbon dots) combined with broad and strong absorption are at the heart of the many present applications of luminescent quantum dots in e.g. displays and bio-imaging. Adding polarization will offer additional benefits, most clearly for LCD displays where already red and green emitting quantum dots are used in QLED televisions. Polarized emission from QDs will strongly increase the display energy efficiency.

The discovery of unique polarization properties of light for 0D/2D hetero nanostructures provides new perspectives and methods for developing luminescent materials with tailor-made polarization dependent optical properties based on new types of nanocomposites. Grafting nanosized 0D emitters on other 2D nanosheets and even 1D nanorods can be explored in this new family of polarized light emitters. In the future, these composite materials not only offer exciting physics but are also expected to have important applications in various fields including displays, photocatalysis, biomedical polarized imaging, optical data storage and optical communication.

## References

[1] Perrin F (1926). Polarisation de la lumière de fluorescence. Vie moyenne des molécules dans l’etat excité. J. Phys. Radium.

[2] Srivastava AK (2017). Photoaligned nanorod enhancement films with polarized emission for liquid-crystal-display applications. Adv. Mater..

[3] Gu M, Zhang QM, Lamon S (2016). Nanomaterials for optical data storage. Nat. Rev. Mater..

[4] Qi L (2017). MnO_2_ nanosheet-assisted ligand-DNA interaction-based fluorescence polarization biosensor for the detection of Ag^+^ ions. Biosens. Bioelectron..

[5] Cao WH (2019). Measuring nanoparticle polarizability using fluorescence microscopy. Nano Lett..

[6] Zhou XP (2023). Narrow-band blue-emitting indium phosphide quantum dots induced by highly active Zn precursor. Adv. Opt. Mater..

[7] Wang BY (2022). Electron–phonon coupling-assisted universal red luminescence of o-phenylenediamine-based carbon dots. Light Sci. Appl..

[8] Ding BF (2020). Giant magneto-birefringence effect and tuneable colouration of 2D crystal suspensions. Nat. Commun..

[9] Xu H (2022). Magnetically tunable and stable deep-ultraviolet birefringent optics using two-dimensional hexagonal boron nitride. Nat. Nanotechnol..

[10] Huang ZY (2022). 2D functional minerals as sustainable materials for magneto-optics. Adv. Mater..

[11] Lan TS (2021). Collective behavior induced highly sensitive magneto-optic effect in 2D inorganic liquid crystals. J. Am. Chem. Soc..

[12] Xu HW (2023). A multifunctional optoelectronic device based on 2D material with wide bandgap. Light Sci. Appl..

